# Improving knowledge and behavior of leadership and followership among the interprofessional team

**DOI:** 10.5116/ijme.5b30.9a84

**Published:** 2018-06-29

**Authors:** Dana Tschannen, Rebecca Dorn, Courtney Tedesco

**Affiliations:** 1 University of Michigan, School of Nursing, USA

**Keywords:** Teamwork, leadership and followership, interprofessional education, communication, virtual training

## Abstract

**Objectives:**

To examine virtual training on Crew Resource Management (CRM) principles of effective leadership and followership on participants’ knowledge, applicability, and intended behaviors.

**Methods:**

Graduate students (n=41) from four health disciplines participated in the training, which included a self-learning module (e.g., didactic content and video vignettes) and an optional virtual simulation exercise. Knowledge was examined via a 10-item pre/post knowledge test. Applicability of the training and intended behaviors was measured post-training via an 11-item survey. T-test and Analysis of variance were applied to compare knowledge scores, as well as to determine variation in discipline responses.

**Results:**

Knowledge improved significantly post-training (t_(40)_=10.47, p<0.001). Pharmacy students scored significantly lower on the post-knowledge test than medicine and nursing students [F_(2,36)_=5.99, p=0.006]. On average, participants completing the module reported learning new skills and knowledge (M=4.17, SD=0.54) and intended to use skills/knowledge gained from the training in clinical practice (M=4.29, SD=0.56). No differences were noted among responses from the various disciplines. Those completing the simulation exercise (n=10) found value in the experience, again noting strong application to practice (M=4.9, SD=0.32) and intended use in practice (M=4.9, SD=0.32).

**Conclusions:**

The CRM training was valuable and applicable to practice. Use of a virtual platform may provide an avenue for minimizing current barriers to successful interprofessional education by allowing participants to connect in various geographical locations. The module is ready for widespread use in health professional education.

## Introduction

Interprofessional education (IPE) aims to prepare health professional students for purposeful, collaborative work towards a common goal of delivering safe, patient-centered care.^1^ Healthcare disciplines—nursing, medicine, pharmacy, public health, social work, dentistry—have identified the need for interprofessional collaboration, teamwork, and effective communication in their respective core competencies and program requirements.^2^^-^^6^ Optimal patient care occurs within a culture that promotes cooperation and open communication within the interprofessional team while integrating patient needs into the plan of care.^2^^-^^6^ In 2016, the Interprofessional Education Collaborative (IPEC) reaffirmed the value and impact of the core competencies originally developed in 2011, which included interprofessional teamwork/team-based practice and interprofessional communication.^1^^,^^7^

Miscommunication continues to be a primary cause of sentinel events despite consensus on the need for effective teams and interprofessional communication.^8^ Strategies designed to improve communication and teamwork within the interprofessional team is needed. Crew Resource Management (CRM), which focuses on the management of human error and risk reduction in the environment, is one training method with unique characteristics that have shown promise in mitigating risks stemming from communication failures in healthcare.^9^

The principles of CRM, with its aviation industry roots, require crew members to identify threats in the environment, communicate those threats to a person in charge, and collectively develop a plan for error mitigation.^10^ A recent meta-analysis showed CRM training to have a large positive effect on participants’ knowledge and behaviors and a small effect on attitudes.^11^ Effective leadership and followership, situational awareness, assertive communication strategies, human factors issues, and checklists are concepts covered in CRM training.

CRM training has shown to improve patient safety behaviors and outcomes, including patient mortality, operating room efficiencies (e.g., delays, handoffs), and medication error rates.^12^^,^^13^ After a system-wide rollout of CRM training (n= 3600 health system employees in 12 areas) in one Midwestern health system, the number of adverse events avoided was 735, a 25.7% reduction in observed relative to expected events. Additionally, the cost savings over three years ranged from $12.6 to $28.0 million, with a return on investment of $9.1 to $24.4 million.^14^^,^^15^ Furthermore, Haerkens and colleagues—in a 3-year cohort study in the intensive care unit—reported a significant decrease in complication rate pre- (67.1/1000 patients) to post-training (50.9/1000 patients) with a corresponding decrease in odds of mortality. The incidence of cardiac arrests also decreased, with cardiac resuscitation success rates increasing from 19% to 67%.

Integrating CRM principles into the educational curriculum of the interdisciplinary team may be what is needed to improve the collaborative work of the healthcare team, subsequently improving patient care. Despite the desire to integrate IPE content related to providing effective teams and communication into health discipline curricula, barriers related to cost, labor, lack of faculty support, and inter-school scheduling conflicts continue to impact the opportunity for joint training.^16^ Staff shortages, location accessibility, and training delivery modalities add to the difficulty of conducting a successful training session.^17^ Additionally, training not directly related to a course for students or required for staff in the healthcare field limit accessibility and willingness to participate.^18^ Standard, didactic, face-to-face training sessions do not allow for the versatility needed to reach the full interpersonal team effectively. For this reason, use of virtual modalities was explored. The purpose of this study was to examine a virtual interprofessional education focused on the CRM principles of effective leadership and followership on participants’ knowledge, practical applicability, and intended behaviors. Specifically, it was hypothesized that CRM knowledge would increase post-training. Additionally, it was believed that participants would recognize the applicability of the CRM tools, reporting intentions to use CRM strategies in their respective practice upon completion of the CRM training.

## Methods

### Study design and participants

This study utilized a pre-post, test design, with a sample of healthcare professionals currently enrolled in graduate studies (e.g., nursing, medicine, pharmacy, and social work) at one Midwestern University. Recruitment for participation in the study occurred through two primary modalities: (1) an email invitation from department faculty in the respective disciplines, and (2) an introductory presentation on CRM from the principal investigator at a jointly sponsored event by the Institute for Healthcare Improvement (IHI) Open School and the Interprofessional Health Student Organization (IHSO) at the respective university. A total of 53 graduate students signed up to complete the CRM module, with 41 students (response rate 77%) completing the module and pre-/post-knowledge test. The majority of participants completing the CRM module were pharmacy students (n=18), followed by medicine (n=15), nursing (n=6), and social work (n=2). Ten students participated in the CRM simulation session, which was an optional component of the CRM training. The affiliated Institutional Review Board deemed the study exempt prior to conduction of the study. The exemption was determined since the study was conducted in an established educational setting involving normal education practices.

### Intervention

The primary competency for the CRM training was for participants to implement effective leadership and followership strategies in the healthcare environment, optimizing patient outcomes and care processes. The CRM training intervention consisted of two components: a self-learning educational module, and a CRM-focused virtual simulation session. Participants had the option of completing both components of the training or just the self-learning module. A group of staff nurses previously evaluated the components of the module and found the training to be very applicable to their work. Specific details regarding the training development and beta testing are reported in detail elsewhere, but highlights are provided here.^18^

CRM Self-Learning Module. The online CRM module was offered through an online learning platform. The CRM training module was comprised of narrated slides and video vignettes containing key CRM concepts related to effective leadership and followership. Specifically, leadership topics included types of leaders, effective leadership skills (e.g., inviting participation, engaging the team, asking team questions), closed-loop communication, huddles, debriefings, and checklists. Effective followership content included types of followers, avoidance of hint and hoping in communication, and a description of the Effective Followership Algorithm, which provides communication tools for escalating communication.^19^ The Effective Followership Algorithm provides healthcare professionals with specific actions that can be taken when his/her viewpoint is not being heard. This begins with the 3Ws (e.g., What I see…, What I am concerned about…, and What I want…). The phrases within the 3Ws provide a script for how to clearly, succinctly communicate key elements in a given clinical scenario. In the event a professional feels the need to escalate the communication further, he/she could use the 4 Step Assertive Tool (e.g., Get Attention, State Concern, Offer Alternative, Pose a Question). Additional actions that can be taken at any time during the escalating situation include engaging the team, taking action, or using the chain of command.

Within the CRM module, the narrated slides provided a brief overview of the CRM concepts, with video vignettes role-playing the CRM concepts in practice. Students received access to the learning module by the principal investigator, with instructions on how to complete the training. The module could be completed in one or multiple sessions, depending on the participant’s preference. Completion of the module was expected to take 60-90 minutes, and all participants were provided an incentive for their participation.

CRM Simulation Session. The CRM virtual simulation—an optional component of the training—was designed to provide participants with an opportunity to practice the CRM skills learned in the module. The virtual platform used for the simulation session was Second Life, an open-access multi-user virtual environment that uses avatars to interact with the environment. Second Life supports a high degree of interactivity, including role-plays and simulations, thus providing a rich environment for multi-disciplinary team training.^20^ A virtual hospital—used in previous research—was used as the background for the simulation session.

During the CRM simulation session, a recap of the key principles of CRM was given, followed by participation in three virtual scenarios requiring participants to use effective leadership or followership skills. Two of the scenarios (Table 1) required participants to use the Effective Followership Algorithm, as the scenario was scripted in a way that required an escalation of communication among the healthcare team. A final scenario required participants to lead a daily huddle for their patients among the interprofessional team. In each scenario, participants played the role of their specific discipline (e.g., the pharmacy student played the role of the pharmacist). The facilitators (e.g., the principal investigator and two graduate students) of the simulation session played the role of the other disciplines as needed. A debrief was conducted after each scenario, asking participants to identify what went well, what could have been done differently, and the applicability of the CRM principles to practice.

Despite the use of a virtual platform, the simulation sessions occurred in the school of nursing to decrease the risk of any technical malfunctions that may have affected the study. One individual completed the training virtually without any difficulty. Each simulation session—conducted over a two-day period—included 1-3 participants from four disciplines. Upon arrival, the facilitator provided an overview of the virtual simulation agenda, and participants moved into separate rooms to simulate an individual off-site learning experience. Each student was equipped with a computer, a microphone headset, and a printout of CRM communication tools. The session lasted approximately 60 minutes, and all participants received an incentive (e.g., an Amazon gift card) for participation.

**Table 1:** Virtual simulation session scenario overviews

Scenario Topic	Scenario Overview
Effective Followership Algorithm	Theme: A new mother with a history of depression is being discharged without proper medications. Safety concerns arise with the child (e.g., no car seat). Expectations: Participant contacts provider to discuss concerns with medication and need for the car seat. Communication escalation required (as scripted).
Effective Followership Algorithm	Theme: A patient with Primary CNS Lymphoma is being treated with high-dose methotrexate and needs to receive medication to increase urine pH. The nurse fails to give the medication required. Expectations: Provider and pharmacist notify the nurse of the needed medication on multiple occasions without a response. Communication escalation required (as scripted).
Huddles/Checklists	Theme: Summary of patients’ stories on a general unit provided. Participants perform a daily huddle with the interprofessional team. Expectations: Participant leads a huddle using a checklist, engaging the interprofessional team in patient care and safety concerns.

### CRM training evaluation

Participants completed an evaluation after each component of the training to examine the effectiveness of the CRM training on knowledge, practical applicability, and intended behaviors. To examine knowledge around CRM, participants completed a 10-point multiple choice pre- and post-test aimed to measure learner understanding of effective leadership and followership principles. Both knowledge tests consisted of knowledge (e.g., At what point in the Effective Followership Algorithm should someone Take Action as his or her next step?) and application questions (e.g., You are in Mr. Smith’s room taking vitals when you see that his blood pressure and heart rate are abnormally high. Which of the following statements best communicates the information in a direct and concise manner?) A correct response resulted in the achievement of one point, for a maximum of 10 points if all responses were correct. The knowledge tests had previously been reviewed for face validity, relevancy, and alignment with CRM content.^18^ The knowledge tests were embedded into the CRM module.

To examine intended behaviors and overall applicability with the CRM training, a survey was given to the participants at the completion of both components of the training. The survey consisted of 11 items asking questions related to training content, teaching strategy, intended use, and applicability to practice.^21^ The majority of items were measured on a 5-point Likert scale from 1 (strongly disagree) to 5 (strongly agree). Three items were open-ended questions aimed at identifying the most beneficial aspect of the training, the best area for improvement, and the content most useful to their practice. The tool had been used in other studies.^18^^,^^21^

Participants completed the survey after each training component. Those completing both aspects of the training (e.g., the module and the simulation) completed the survey twice, reflecting on their experience with each component of the training specialty.

### Data analysis

Data were analyzed using SPSS® (Chicago, IL) Version 24.0 for Windows® software. Computations for means, ranges, and standard deviations were completed. To determine the effectiveness of the CRM training, a paired t-test was used to compare knowledge scores pre- and post-training. Additionally, comparisons of knowledge means were made between the various disciplines using Analysis of variance (ANOVA). The Post-hoc tests that were performed used Bonferroni correction. Additionally, an ANOVA was conducted to compare discipline responses to the effectiveness of the training (e.g., intended behaviors, practice applicability). Confidence intervals were set at 95% and a p-value of < .05 was considered statistically significant. Due to a very small sample of social work students completing the training, data from these students were removed for the inferential statistical analysis evaluating discipline variation.

## Results

### Knowledge

Pretest knowledge score, for all disciplines, had a mean score of 7.07 (SD=1.57), with a range of 3-10 (Table 2). Social work students had the lowest pre-test knowledge score, followed by the pharmacy, nursing, and medical students. ANOVA identified no significant variations between the disciplines for the pre-knowledge test scores (F_(__2, 36)_ =2.05, p=0.14).

Post-knowledge test scores improved as evidenced by a mean score of 9.63 (SD=0.58) for all disciplines, with a range of 8-10. Nurses received a perfect post-test score mean of 10.0, followed by improved scores from medicine, social work, and pharmacy. The paired t-test was used to compare the mean scores of the pre-test (7.07±1.57) and post-test (9.63±0.58), which showed significant improvements in knowledge [t_(__40)_=10.47, p<.001]. Furthermore, ANOVA showed a significant difference in the post-knowledge scores between disciplines [F_(__2,36)_ = 5.99, p=.006]. Specifically, a significant difference was noted between pharmacy students and both medicine (p=.017) and nursing students (p=.03). No difference was noted between nursing and medicine students (p=1.0).

### Applicability and intended behaviors—CRM module

Participants were asked to examine the applicability, and intended use of skills learned in the CRM module via a post-training survey (Table 3). The CRM concepts applied to patient care as a way to reduce harm (4.59 ± 0.5). Students reported learning new skills and knowledge from the module (4.17 ± 0.53) and intended to use the skills/knowledge gained from the training in clinical practice (4.29 ± 0.56). The CRM module was noted to be worthwhile (4.27 ± 0.59) and students thought the teaching strategies were effective (4.13 ± 0.83). Overall, students recommended this module to other clinicians (4.32 ± 0.72) and indicated they were interested in more training similar to the CRM training (3.9 ± 0.70). In response to the question related to the content most useful to practice, the respondents denoted content related to closed loop communication (e.g., communication receiver repeats back the sender’s words) and the Effective Followership Algorithm.

**Table 2:** CRM module pre- and post- knowledge test scores by discipline

Discipline	Test	Mean	SD	CI
Medicine	Pre-test	7.73	1.03	7.16, 8.31
	Post-test	9.87	.35	9.67, 10.06
Nursing	Pre-test	7.17	1.33	5.77, 8.56
	Post-test	10.0	0.0	10.0, 10.0
Pharmacy^*^	Pre-test	6.72	1.71	5.87, 7.57
	Post-test	9.33	.69	8.99, 9.67
Social Work	Pre-test	5.0	2.0	-20.41, 30.41
	Post-test	9.5	0.5	3.15, 15.85
Total^**^	Pre-test	7.07	1.57	6.58, 7.57
	Post-test	9.63	0.58	9.45, 9.82

Note: Social work students were removed from the discipline specific comparisons.

*ANOVA noted a significant difference in post-tests by discipline [F(2,36)=5.99, p= .006]. Pharmacy students scored significantly less post-test than both medicine (p=.017) and nursing students (p=.03).

**Paired t-test noted significant improvements in knowledge pre- to post-test [t(40)= 10.47, p<.001].

Discipline-specific feedback on the CRM module was also examined (Table 3). Nurses found the concepts to be most applicable to care (5.0 ± 0.0) and saw themselves using the skills/knowledge gained in the session in clinical practice (4.67±0.52) more than any other discipline. Additionally, nurses recommended the training to other clinicians and were interested in more training similar to CRM (4.17 ± 0.41). The social work student reported the highest mean associated with developing new skills/knowledge (4.5±0.71) and acknowledged use of effective teaching strategies in the module (5.0±0.0). Pharmacy students reported the CRM training to be the most worthwhile (4.39 ± 0.61) over all other disciplines. Although scores remained high (e.g., above four on all but one item), medical students rated each aspect of the training the lowest among the disciplines (e.g., the applicability of the CRM principles, development of new skills/knowledge and use of these skills, the recommendation of training to others, etc.). Despite this, ANOVA analysis revealed no significant differences among the disciplines’ response to CRM module applicability and intended behaviors.

**Table 3:** CRM Module evaluation survey by discipline

Items	All (Mean ± SD)	Medicine (Mean ± SD)	Nursing (Mean ± SD)	Pharmacy (Mean ± SD)	p-value
The CRM concepts can be applied to patient care to reduce harm to patients.	4.59±0.5	4.53±.52	5.0±0.0	4.5±0.51	.09
I developed new skills and/or knowledge as a result of my participation in the CRM session.	4.17±0.53	4.0±0.53	4.17±.41	4.28±0.57	.35
I see myself using the skills and/or knowledge gained from the CRM session in the clinical area.	4.29±0.56	4.07±0.59	4.67±0.52	4.33±0.49	.07
Overall, the CRM session was worthwhile.	4.27 ± 0.59	4.13±0.64	4.33±0.52	4.39±0.61	.48
The teaching strategies used in this session were effective.	4.13±0.83	4.07±0.88	4.4±0.55	4.11±0.90	.75
I would recommend this training for other clinicians.	4.32±0.72	4±0.93	4.5±0.55	4.44±0.51	.16
I would be interested in more training that is similar to this training.	3.9±0.70	3.6±0.83	4.17±0.41	4.06±0.64	.12

Note: Social Work student responses removed for the inferential analysis

In a free text response to what the students liked best about the module training, several students accredited how the CRM concepts were presented. This included comments such as “the video examples were helpful to see the theories in action (No 1, social work student)” and “the powerpoint slide lectures were concise and easy to follow which helped with retaining the most important elements (No 2, pharmacy student).” Several respondents also emphasized the applicability to real life, as evidenced by this comment: “I enjoyed the example situations that were specific to my discipline and I thought they were realistic (No 3, pharmacy student).” Some areas for improvement that the students cited included the amount of time the module took and the lack of interaction, stating, “Some of the powerpoint style videos were too long/too many (No 4, medical student)” and “more questions/quizzes interspersed would be good to check understanding and make it more interactive (No 5, pharmacy student).”

### Applicability and intended behaviors—CRM simulation training

Students from three different disciplines participated in the simulation: pharmacy (n=7), nursing (n = 2), and social work (n = 1). Due to the very small sample, only descriptive statistics were computed (Table 4). Similar to the CRM module feedback, the students strongly believed the CRM simulation session was worthwhile (4.9 ± 0.32) and believed they developed new skills and knowledge (4.9±0.32). They also strongly believed that the CRM concepts applied to patient care (4.9 ± 0.32) and could see themselves using these skills in the clinical setting (4.9 ± 0.32). The respondents unanimously and strongly agreed that the teaching strategies were effective, and that they would recommend this training to other clinicians (5± 0.0). They also strongly agreed they would be interested in more training similar to this (4.9 ± 0.32).

One aspect the respondents liked about the simulation training session was the interactive nature, as noted by one learner who stated that “being virtual was much more interactive than the video modules and that was helpful for practicing and solidifying the techniques (No 1, student).” They also appreciated the applicability to clinical practice, commenting that “the role-playing was more realistic (No 4, student).” An area the respondents cited for improvement was more of an emphasis on increasing interprofessional participation, suggesting it be “incorporated into the IPE course (No 5, student).”

**Table 4:** CRM simulation evaluation survey

Items	Mean ± SD
The CRM concepts can be applied to patient care to reduce harm to patients.	4.9±0.32
I developed new skills and/or knowledge as a result of my participation in the CRM session.	4.9±0.32
I see myself using the skills and/or knowledge gained from the CRM session in the clinical area.	4.9±0.32
Overall, the CRM session was worthwhile.	4.9±0.32
The teaching strategies used in this session were effective.	5.0±0.0
I would recommend this training for other clinicians.	5.0±0.0
I would be interested in more training that is similar to this training.	4.9±0.32

## Discussion

The purpose of this study was to examine the effectiveness of a virtual interprofessional education focused on the CRM principles of effective leadership and followership on participant’s knowledge, practical applicability, and intended behaviors. It was hypothesized that CRM knowledge would increase post training. This hypothesis was fully supported, as improvements in knowledge of effective leadership and followership were significant post-CRM training. This finding is similar to several other studies that have noted improved knowledge among participants.^22^^,^^23^ The most significant improvement of knowledge was noted for social work students. This may be because they do not receive specific training on effective leadership/followership, or it may be that social work students initially did not see themselves as ‘leaders’. Nurses had the second highest improvement in knowledge gained. This, in part, may be because current nursing curriculum focuses on effective leadership, but not how to be an effective follower (e.g., avoiding hinting and hoping, and recognizing the pivotal role that followers have inpatient care). Additionally, the CRM training provides nurses (and other disciplines) with tools to succinctly communicate escalating

concerns regarding a patient’s status. Pharmacy students—despite improvements in knowledge post CRM training—had a significantly lower post-test score than nurses and medical students. This may be related to their education (e.g., limited curriculum focused on leadership/followership) and perceived practice role. An objective of CRM is for all healthcare professionals to recognize the significant role they play in practice, which requires situations for leadership and followership. When the full interprofessional team integrates CRM principles (e.g., effective leadership and followership), improvements in patient outcomes result.^12^^,^^13^

The second hypothesis stated that learners would recognize the applicability of the CRM tools to practice, having intentions to incorporate CRM strategies into their clinical setting upon completion of the CRM training. Post-evaluation supported this hypothesis as students strongly agreed that the CRM training was worthwhile and applicable to practice. Students also reported their intention to implement CRM training in their practice. This finding is in line with previous studies that reported behavior change post-training.^24^^,^^25^ The teaching strategies—which were also highly rated—took advantage of a virtual platform as a means to engage all members of the healthcare team. The video vignettes were valued, and students liked the opportunity to ‘see’ the CRM concepts in real life scenarios. Additionally, the simulation session provided a safe place for students to practice their skills. Several students commented on the realism of the simulations and liked the opportunity to practice their newly acquired skills. Further studies need to truly determine how this type of training translates into practice for the interprofessional team. Specifically, studies should examine the use of CRM principles and strategies through observation in the clinical setting to determine the effect of CRM training on behavior.

Variations in feedback from the disciplines existed but were not significant. Despite the high ratings from all disciplines, medical students reported lower applicability and worth of the training as compared to all other disciplines. Chan and colleagues found similar findings after implementing CRM training, in that nurses rated the overall usefulness, applicability, and satisfaction higher than their medical counterparts.^27^ In many instances, physicians play the role of team lead during patient care. Medical students have reported themselves as being fully competent in communication (90%), and conflict management (70%), which means the need for CRM training (e.g., effective followership) may not be clearly recognized.^28^ In contrast, other disciplines may see the need for CRM tools such as the Effective Followership Algorithm as beneficial for escalating communication concerns within the interprofessional team.

Clear and concise communication in a deteriorating patient situation can be difficult. The participants identified the Effective Followership Algorithm as the most useful aspect of the training. Healthcare professionals desire tools—rather than theoretical concepts—that are useful in practice. The algorithm provides a step-by-step process for effectively and efficiently articulating essential information during critical patient situations.

Those participating in the CRM simulation session reported very high levels of applicability and value, finding the session to be worthwhile and an excellent opportunity to practice use of CRM principles. Engaging the participant directly through role play allows them to engage in the learning experience fully and provides an opportunity to practice newly-learned skills. Further studies need to assess how experience with simulation may transfer into actual behaviors in the clinical setting. Specifically, does allowing learners to practice leadership and followership skills in a safe environment (e.g., simulation) result in more confidence and subsequent use of CRM tools in the clinical setting?

Several limitations to the study are noteworthy. The study design did not include randomization or a control group. This poses threats to internal validity that must be considered when interpreting the findings. Generalizability is limited since the sample size was relatively small, with discipline-specific samples varying greatly. This warrants further study with larger samples of all members of the interprofessional team. Understanding the variations in discipline feedback may assist with further enhancing the IPE experience. For example, the CRM simulation scenario focused on the daily huddle/briefing by nurses and was thus framed in a manner that made sense to nursing workflow (e.g., beginning of shift, meeting with the nursing staff/team). That same workflow may not be feasible for other disciplines. Revisions to the simulations are needed to incorporate more discipline-specific engagement. A further limitation exists due to the fact that there was only a small number of participants; thus, generalizability is limited. Regression to the mean must also be considered as the participants may have simply had a higher ‘average’ score than if a random sample of students had been identified. Additionally, only a small number of participants completed both aspects of the training. Thus, applicability and value of the CRM training might have improved had both aspects of the training been completed. Those that did participate in the simulation session were very positive about the experience, noting very high degrees of value and applicability.

## Conclusions

Effective communication and teamwork among the interprofessional team are essential to optimal care. IPE is an avenue to create a platform for deliberate cross-discipline training, where students are required to work collaboratively towards high-quality care. Effective teamwork and communication are core competencies in all healthcare disciplines; therefore, strategies that minimize barriers to IPE must be developed. CRM training has resulted in improved team knowledge and behavior, resulting in high-quality care. In this study, knowledge improved significantly, with high levels of agreement in the applicability and intended use of CRM tools in the clinical setting post-training. Communication among the interprofessional team is paramount for high quality care. Utilizing the tools and strategies provided by this CRM training could result in improved teamwork and patient outcomes. Further studies are warranted to examine utilization of CRM principles in practice and their effect on professional and patient outcomes.

Engaging the healthcare team in any interprofessional education is difficult, due to variations in schedules, job responsibilities, and workflow within healthcare. The CRM training module evaluated in this study, which utilized a virtual platform, allowed for engagement without the necessity of being face-to-face in a given location. This format allows for engagement of multiple disciplines without the need of geographical proximity; thus, scaling of the training for widespread dissemination will be relatively simple and cost-effective.

### Acknowledgements

The support for this project comes from the Center for Research on Learning and Teaching at the University of Michigan.

### Conflict of Interest

The authors declare that they have no conflict of interest.

## References

[1] Interprofessional Education Collaborative Expert Panel. Core competencies for interprofessional collaborative practice: report of an expert panel. Washington DC: Interprofessional Education Collaborative; 2011.

[2] American Association of Colleges of Nursing (AACN). The essentials of master's education in nursing [cited 2011 March 21]; Available from: http://www.aacnnursing.org/Portals/42/Publications/MastersEssetials11.pdf.

[3] Accreditation Council for Graduate Medical Education. ACGME common program requirements [cited 2017 July 1]. Available from: http://www.acgme.org/acgmeweb/Portals/O/PFAssets/ProgramRequirements /CPRs07012015.pdf.

[4] American Dental Education Association. Competencies for the new general dentist. J Dent Educ. 2008;72(7):823-826.

[5] Medina MS, Plaza CM, Stowe CD, Robinson ET, DeLander G, Beck DE, Melchert RB, Supernaw RB, Roche VF, Gleason BL, Strong MN, Bain A, Meyer GE, Dong BJ, Rochon J, Johnston P (2013). Center for the Advancement of Pharmacy Education 2013 educational outcomes.. Am J Pharm Educ.

[6] Accreditation Council for Graduate Medical Education. ACGME common program requirements [cited 01 July 2017]; Available from: https://s3.amazonaws.com/aspph-wp-production/app/uploads/2014/04/Version2.31_FINAL.pdf.

[7] Interprofessional Education Collaborative (IPEC). Core competencies for interprofessional collaborative practice: 2016 update. Washington DC: Interprofessional Education Collaborative; 2016.

[8] The Joint Commission. Sentinel event data-root causes by event type 2004.Oakbrook Terrace, IL: The Joint Commission; 2

[9] Sculli GL, Fore AM, Neily J, Mills PD, Sine DM (2011). The case for training Veterans Administration frontline nurses in crew resource management.. J Nurs Adm.

[10] Tschannen D, McClish D, Aebersold M, Rohde JM (2015). Targeted communication intervention using nursing crew resource management principles.. J Nurs Care Qual.

[11] O'Dea A, O'Connor P, Keogh I (2014). A meta-analysis of the effectiveness of crew resource management training in acute care domains.. Postgrad Med J.

[12] Neily J, Mills PD, Young-Xu Y, Carney BT, West P, Berger DH, Mazzia LM, Paull DE, Bagian JP (2010). Association between implementation of a medical team training program and surgical mortality.. JAMA.

[13] Fore AM, Sculli GL, Albee D, Neily J (2013). Improving patient safety using the sterile cockpit principle during medication administration: a collaborative, unit-based project.. J Nurs Manag.

[14] Moffatt-Bruce SD, Hefner JL, Mekhjian H, McAlearney JS, Latimer T, Ellison C, McAlearney AS (2017). What is the return on investment for implementation of a crew resource management program at an academic medical center?. Am J Med Qual.

[15] Haerkens MH, Kox M, Lemson J, Houterman S, van der Hoeven JG, Pickkers P (2015). Crew resource management in the intensive care unit: a prospective 3-year cohort study.. Acta Anaesthesiol Scand.

[16] Oandasan I, Reeves S (2005). Key elements of interprofessional education. Part 2: factors, processes and outcomes.. J Interprof Care.

[17] Ward J, Wood C (2000). Education and training of healthcare staff: the barriers to its success.. Eur J Cancer Care (Engl).

[18] Tschannen D (2017). Development of a virtual crew resource management training program to improve communication.. J Contin Educ Nurs.

[19] Sculli G, Sine D. Soaring to success: taking crew resource management from the cockpit to the nursing unit. Danvers, MA: HCPro, Inc.; 2011.

[20] Boulos MN, Hetherington L, Wheeler S (2007). Second Life: an overview of the potential of 3-D virtual worlds in medical and health education.. Health Info Libr J.

[21] Aebersold M, Tschannen D, Sculli G (2013). Improving nursing students' communication skills using crew resource management strategies.. J Nurs Educ.

[22] Pettker CM, Thung SF, Raab CA, Donohue KP, Copel JA, Lockwood CJ, Funai EF (2011). A comprehensive obstetrics patient safety program improves safety climate and culture.. Am J Obstet Gynecol.

[23] Sculli GL, Fore AM, West P, Neily J, Mills PD, Paull DE (2013). Nursing crew resource management: a follow-up report from the Veterans Health Administration.. J Nurs Adm.

[24] Katheria A, Rich W, Finer N (2013). Development of a strategic process using checklists to facilitate team preparation and improve communication during neonatal resuscitation.. Resuscitation.

[25] Verbeek-van Noord I, de Bruijne MC, Twisk JW, van Dyck C, Wagner C (2015). More explicit communication after classroom-based crew resource management training: results of a pragmatic trial.. J Eval Clin Pract.

[26] Antonacci D, DiBartolo S, Edwards N, Fritch K, McMullen B, Murch-Shafer R. The power of virtual worlds in education: a second life primer and resource for exploring the potential of virtual worlds to impact teaching and learning. ANGEL Learning [cited 01 July 2008]; Available from: http://www.angellearning.com/products/secondlife/downloads/The%20Power%20of%20Virtual%20Worlds%20in%20Education_0708.pdf.

[27] Chan CK, So HK, Ng WY, Chan PK, Ma WL, Chan KL, Leung SH, Ho LY (2016). Does classroom-based crew resource management training have an effect on attitudes between doctors and nurses?. Int J Med Educ.

[28] Varkey P, Peloquin J, Reed D, Lindor K, Harris I (2009). Leadership curriculum in undergraduate medical education: a study of student and faculty perspectives.. Med Teach.

